# Squamous Cell Carcinoma of Duodenum Secondary to Metastasis From Recurrent Head and Neck Cancer: A Case Report and Literature Review

**DOI:** 10.7759/cureus.38898

**Published:** 2023-05-11

**Authors:** Shin T Zaw, Thinzar Zaw, Ri Chen, Ahmad El-Far

**Affiliations:** 1 College of Osteopathic Medicine, Lake Erie College of Osteopathic Medicine, Bradenton, USA; 2 College of Medicine, University of Central Florida College of Medicine, Orlando, USA; 3 Hematology and Oncology, Winter Haven Hospital, Winter Haven, USA

**Keywords:** recurrent cancer, tonsillar squamous cell carcinoma, head and neck oncology, squamous cell carcinoma, metastatic squamous cell carcinoma

## Abstract

This report describes a case involving the diagnosis and treatment of squamous cell carcinoma (SCC) of the duodenum, which was found to be a metastatic lesion originating from recurrent head and neck cancer (HNC) in a 74-year-old female patient. The patient had a past medical history of gastroesophageal reflux disease (GERD), tonsillar SCC, and recurrent HNC. She presented with symptoms of burning, tingling, and numbness of the throat and left side of the tongue. Upon examination with an esophagogastroduodenoscopy, an ulcerated hard area mass was detected in the third portion of the duodenum. Biopsy results confirmed the mass to be a metastatic poorly differentiated SCC.

The incidence of head and neck squamous cell carcinoma (HNSCC) metastasis to the duodenum is rare, likely due to the unique anatomic location and the lack of lymphatic drainage in the area. The patient was treated with a combination of paclitaxel, carboplatin, and pembrolizumab. This case underscores the significance of considering unusual sites of metastasis in HNSCC patients and utilizing advanced imaging modalities and immunotherapy to detect and treat these locations effectively.

## Introduction

Head and neck squamous cell carcinoma (HNSCC) is a widespread malignancy, ranking as the sixth most common cancer worldwide. In 2018, there were 890,000 new cases and 450,000 deaths attributed to HNSCC. Unfortunately, the prevalence of this condition is expected to grow significantly, with an estimated 1.08 million new cases annually by 2030 [1]. While the larynx and pharynx are the primary sites of squamous cell carcinoma (SCC), it can also occur in other regions, such as the oral and nasal cavities and sinuses. SCC has a bleak prognosis, with a five-year survival rate ranging from only 55% to 66% [2]. The development of distant metastases is a significant prognostic factor, with an overall five-year survival rate of less than 10% [3-5]. HNSCC most commonly metastasizes to the lungs, followed by the bone and liver. Although it is a rare occurrence, HNSCC can spread to the GI tract and small intestine. Specifically, there have been only a few reported cases of HNSCC metastasizing to the duodenum, emphasizing the rarity of this condition [6,7]. This case report aims to detail the presentation, diagnosis, and management of a patient with SCC of the duodenum, which was secondary to metastasis from recurrent head and neck cancer (HNC). Additionally, it aims to discuss the implications of the findings in relation to the current literature.

## Case presentation

The patient is a 74-year-old female with a past medical history of gastroesophageal reflux disease (GERD), tonsillar SCC, and recurrent HNC presented for burning, tingling, and numbness of the throat and left side of her tongue. She reports she has no appetite and has not been eating due to difficulty swallowing food. Her family history is unremarkable. She is a former pack-a-day smoker of 25 years.

In 2017, the patient presented for "mouth sores" and had a tonsillectomy and PET scan performed. Histopathology from the tonsillectomy indicated a 2.5 x 1.5 x 1 cm tumor, invasive squamous, with perineurial invasion, and p16 positive. PET scan showed hypermetabolic activity in the left tonsillar area and left neck lymph nodes. The patient was staged with T2M2N0 and stage IVa disease. For six months, the patient underwent weekly treatment with combined chemoradiotherapy using carboplatin and paclitaxel. This treatment approach resulted in the patient achieving complete remission of her disease.

In 2019, the patient experienced cancer recurrence in the retropharyngeal area. The treatment approach involved administering chemotherapy medications, carboplatin, and paclitaxel, alongside the immunotherapy drug pembrolizumab for five cycles. After completing the initial treatment regimen, the patient continued to receive immunotherapy with pembrolizumab for a year. The treatment proved to be effective, and the patient achieved complete remission.

In 2021, a PET scan revealed a right level II disease recurrence. The patient was treated with cetuximab for a total of 12 sessions, but a follow-up PET scan indicated that the disease had progressed. Consequently, the patient underwent a salvage right neck dissection, which involved a maximum resection of the tumor as it had adhered to tissues, including bone and blood vessels.

**Figure 1:**
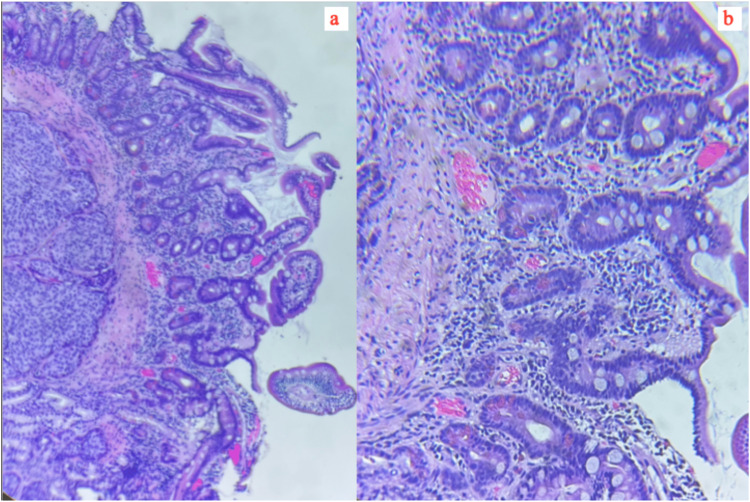
Histopathological image of duodenal SCC (H&E stain (a) 4x and (b) 40x)

In 2022, a CT and PET scan revealed the emergence of small pulmonary nodules that measured less than a centimeter in diameter. These nodules displayed abnormal elevated metabolic activity, and there was also evidence of abnormal metabolic activity within the mediastinal, right hilar, and right supraclavicular nodes. Furthermore, there was an observed increase in the size of nodular opacities located in the peripheral pleural and subpleural regions. As the patient complained of dysphagia, gastroenterology was consulted, and an esophagogastroduodenoscopy to the fourth portion of the duodenum was performed. An ulcerated hard mass was seen in the third portion of the duodenum, and several biopsies were obtained. Subsequent histopathological analysis of the biopsy specimens revealed the presence of metastatic, poorly differentiated SCC cells after being subjected to hematoxylin and eosin (H&E) staining (Figure 1). Further analysis revealed that the tumor cells tested positive for cytokeratin AE 1/3 and p63 while testing negative for CK7, CK20, CDX2, synaptophysin, chromogranin, CA19-9, and CD56. The patient received six cycles of treatment with the regimen of paclitaxel, carboplatin, and pembrolizumab, and imaging showed stable conditions for two months following the completion of treatment. The patient was recommended to start durvalumab for maintenance therapy, but she has decided not to pursue further therapy and has entered hospice care.

## Discussion

This case report draws attention to the infrequent occurrence of metastasis to the duodenum in patients with HNSCC. The literature shows that HNSCC typically metastasizes to the lung, followed by the bone and liver [3-5]. Metastasis to the GI tract, particularly the duodenum, is a rare phenomenon with limited documented cases available [6,7]. A comprehensive literature search was conducted on PubMed and Google Scholar utilizing key terms such as "small bowel metastasis," "duodenal metastasis in head-and-neck cancers," "head-and-neck squamous cell carcinoma," and "HSNCC." Additional medical subject headings (MeSH) terms, such as "metastatic duodenal carcinoma" and "head-and-neck cancer," were also incorporated to ensure a more exhaustive search. Only publications related to duodenal metastasis in HNSCC were included in the analysis, while publications mentioning intestinal metastasis from other HNCs were excluded. Furthermore, the reference lists of relevant articles were thoroughly examined, and any additional pertinent works were added to the review. Overall, 17 cases that met the inclusion and exclusion criteria were identified, with this case report being the 18th (Table 1) [6-22]. Of these reported cases, only six were identified as duodenal metastases, while the remaining cases consisted of eight ileal metastases, three jejunal metastases, and one case that did not specify the precise location of the metastasis.

**Table 1:** Reported cases of HNSCC that metastasized to the small bowel HNSCC: head and neck squamous cell carcinoma

Author	Age/sex	Site of primary presentation	Stage of HNSCC at diagnosis	Presenting symptoms	Time interval between the primary tumor and intestinal metastasis	Site of metastasis	Treatment	Outcome
Boquien et al. [8]	84 male	Pyriform recess	Not reported	Obstruction	10 months	Not reported	Resection anastomosis	Post-surgical death, after 15 days
Bresler et al. [9]	70 male	Supraglottic larynx	T3N1M1	Obstruction	Two days	Ileum	Resection anastomosis	Post-surgical death due to respiratory distress, after seven days
Lesur et al. [10]	90 male	Tonsillar carcinoma	Not reported	Obstruction	Three months	Ileum	Resection anastomosis	Died one month postoperatively due to pulmonary embolism
François et al. [11]	59 male	Supraglottic larynx	T2N2Mx	Intestinal bleeding	29 months	Ileum	Resection anastomosis	Not reported
Petiot et al. [12]	78 male	Supraglottic larynx	T1NxMx	Perforation	12 months	Jejunum	Resection anastomosis	Lived for three months
Hamdan et al. [13]	68 male	Larynx	Not reported	Perforation	36 months	Ileum	Resection and jejunostomy	Died three days after surgery due to septic shock
Airoldi et al. [14]	54 male	Supraglottic larynx	T3N1M0	Intestinal bleeding	18 months	Ileum	Resection anastomosis	Died 10 months later due to the progression of local disease in the head and neck
Gonzales et al. [15]	72 male	Vocal fold	T3N2M0	Perforation	Not reported	Jejunum	Resection anastomosis	Died after 14 days postoperatively because of respiratory distress
Yoshihara et al. [16]	71 male	Supraglottic larynx	T4N0	Obstruction	24 months	Ileum	Resection anastomosis	Died after six months because of abdominal recurrence
Büyükçelik et al. [17]	71 male	Vocal fold	Not reported	Biliary obstruction	56 months	Duodenum	Endobiliary stenting and palliative CT	Due to the progression of the disease, died in 12 months
Guillem et al. [18]	63 male	Base of tongue	T4N1M1	Obstruction	Since initial presentation	Ileum	Resection anastomosis	Died after eight months because of abdominal recurrence
Dwivedi et al. [6]	65 male	Base of tongue	T4N2cM0	Intestinal bleeding	10 months	Jejunum	Palliative CT	Died after one month due to chest infection
Ando et al. [19]	71 male	Supraglottic larynx	T4N2M0 stage IV	Obstruction	13 months	Duodenum	Endoscopic duodenal stenting	The patient was recovering after three months of treatment at the time of reporting of case
Tarangelo et al. [20]	65 male	Right Tonsil, tongue & pharynx	Not reported	Melaena	Six months	Duodenum	Home hospice	Not reported
Ahmed and Lenza [7]	68 male	Right aryepiglottic fold and vallecula	Not reported	Abdominal pain	Three months	Duodenum	Home hospice	Patient died within one month of discharge from hospital
Rajesh et al. [21]	60 male	Tonsil	Not reported	Abdominal pain	Three months	Duodenum	Not reported	Not reported
AlOmran et al. [22]	76 female	Tongue	Stage IVb (T3N2cM0)	Abdominal pain	Seven months	Ileum	Resection anastomosis	Died on day 16 post operation
Our observation	74 female	Tonsil	T2M2N0, stage Iva	Dysphagia	60 months	Duodenum	Chemotherapy	Hospice

While the pathophysiology of duodenal metastasis from HNSCC is not well understood, multiple mechanisms have been identified by which metastases can spread to the small intestine. These include peritoneal dissemination, direct spread from intraabdominal cancer, and hematogenous and lymphatic spread [23-25]. In this particular case, it was hypothesized that the metastasis resulted from direct invasion from the primary tumor site in the larynx. Typical treatment options for duodenal metastasis from HNSCC include surgical resection and systemic chemotherapy, although the individual patient's prognosis depends heavily on the cancer stage at diagnosis.

HNSCC has a well-established poor prognosis, with a five-year survival rate ranging from 55% to 66% [2]. The survival rate is even lower, at less than 10%, when the disease progresses to the point of distant metastases [3-5]. In this case, the patient's prognosis is significantly compromised by metastasis in the duodenum, further reducing their chances of recovery.

The infrequent occurrence of HNSCC metastasis in the duodenum can be attributed to the unique anatomical location of this region, which has limited lymphatic drainage and is less vulnerable to metastasis [6]. However, it is worth noting that the patient, in this case, had a history of recurrent HNSCC and received multiple treatments. While these treatments are intended to target and eliminate cancer cells, they can also have unintended effects that promote cancer growth and spread. For instance, some treatments can induce genetic mutations or weaken the immune system, creating an environment more favorable for metastasis [26]. As a result, the patient's history of cancer and previous treatments may have played a role in the development of distant metastasis to the duodenum.

Despite the rarity of HNSCC metastasis to the duodenum, it is important for healthcare providers to be aware of the potential for rare sites of metastasis in HNSCC and to consider the use of advanced imaging modalities such as PET scans to detect these sites. Additionally, immunotherapy, such as pembrolizumab in combination with chemotherapy, may also be considered a treatment option for patients with HNSCC metastasis to the duodenum [27].

## Conclusions

HNSCC is a common malignancy with a poor prognosis and high mortality rate. While HNSCC metastasis to the duodenum is rare, it is likely due to the unique anatomical location of this region and its limited lymphatic drainage. This case report underscores the importance of recognizing unusual sites of metastasis in HNSCC and the value of advanced imaging techniques and immunotherapy for identifying and treating such sites. In this particular case, the patient had a history of recurrent HNSCC and had received multiple treatments, which could have contributed to the development of distant metastasis. Healthcare providers must remain vigilant for potential rare sites of metastasis in HNSCC, enabling timely diagnosis and treatment to improve the patient's chances of survival.
